# Personalized Selection of Inferior Turbinate Surgery Based on Structural Phenotyping: A Structured Narrative Review and Proposed Decision-Making Framework

**DOI:** 10.3390/jpm16060310

**Published:** 2026-06-08

**Authors:** Alessia Pennacchi, Basile N. Landis, Michael B. Soyka, Roberto Spasiano, Matteo Trimarchi

**Affiliations:** 1 Department of Otorhinolaryngology Head & Neck Surgery, Cantonal Hospital Lugano, 6900 Lugano, Switzerland; alessia.pennacchi@eoc.ch (A.P.); roberto.spasiano@eoc.ch (R.S.); 2 Rhinology-Olfactology Unit, Department of Otorhinolaryngology–Head and Neck Surgery, Geneva University Hospitals, University of Geneva, 1211 Geneva, Switzerland; basile.landis@hug.ch; 3 Department of Otorhinolaryngology, Head and Neck Surgery, University Hospital Zurich, University of Zurich, 8091 Zurich, Switzerland; michael.soyka@usz.ch; 4 Faculty of Biomedical Sciences, Università della Svizzera Italiana, 6900 Lugano, Switzerland

**Keywords:** inferior turbinate hypertrophy, nasal obstruction, turbinoplasty, personalized medicine, phenotype-guided surgery, patient-tailored surgery, nasal valve

## Abstract

**Background:** Inferior turbinate hypertrophy is a major cause of chronic nasal obstruction and can be treated with several surgical techniques. However, current surgical decision-making is often not personalized to the dominant anatomical and functional substrate of obstruction. No widely adopted structural classification of the inferior turbinate exists, and no standardized algorithm links individual anatomical phenotypes to targeted surgical strategies. **Methods:** A structured narrative review of PubMed and Scopus was performed from database inception to 1 February 2026, using predefined search terms and eligibility criteria. Studies were selected if they addressed inferior turbinate anatomy, histopathology, imaging morphology, endoscopic grading, nasal valve or septal anatomy, surgical techniques, postoperative outcomes, complications, or patient-reported outcomes. Randomized and prospective trials, histopathological studies, CT morphometric analyses, and validated endoscopic grading systems were considered. Four phenotypes of inferior turbinate hypertrophy were identified and linked to preferred surgical options within a clinically oriented decision algorithm integrating endoscopy, functional testing, and selective CT imaging. This framework was developed to support individualized treatment planning and shared decision-making. **Results:** Four structural phenotypes were defined: (i) predominantly cavernous/mucosal hypertrophy; (ii) predominantly bony hypertrophy; (iii) anterior nasal valve-turbinate conflict; and (iv) mixed hypertrophy. For mucosal-dominant disease, radiofrequency ablation and laser turbinoplasty are preferred first-line, mucosa-preserving options. For bony hypertrophy, mucosa-preserving powered inferior turbinoplasty is favored for the mid/posterior turbinate, whereas endoscopic pyriform aperture turbinoplasty is preferred for anterior valve-level conflict. Mixed phenotypes are best managed with combined skeletal and mucosal procedures. The algorithm aims to avoid mismatched treatments, such as mucosal-only techniques for rigid bony hypertrophy or extensive skeletal reduction in purely mucosal disease. Perioperative variables relevant to shared decision-making, including type of anesthesia, postoperative morbidity, recovery profile, and expected limitations, were summarized for each technique. **Conclusions:** This phenotype-guided algorithm provides a structured, evidence-informed framework for the personalized selection of inferior turbinate surgery, emphasizing mucosal preservation, anatomical specificity, patient-centered decision-making, and avoidance of mismatched procedures. It is intended to support, not replace, clinical judgment and to guide future prospective validation studies in personalized rhinologic surgery.

## 1. Introduction

Inferior turbinate hypertrophy is one of the most common etiologies of chronic nasal obstruction in both general and specialist otorhinolaryngology practice. Patients frequently report longstanding nasal blockage, sleep disturbance, mouth breathing, reduced exercise tolerance, snoring, and impaired quality of life. The inferior turbinate plays a pivotal role in modulating airflow resistance, humidifying and warming inspired air, and contributing to mucociliary clearance and local immune defense. Consequently, pathology affecting this structure may have disproportionate functional consequences [1,2].

The inferior turbinate is anatomically complex. It consists of an osseous core formed by the inferior concha bone, covered by a thick mucosal layer that contains a rich venous cavernous plexus, seromucinous glands, and submucosal connective tissue. The erectile tissue is capable of marked volume changes, driven by autonomic regulation and inflammatory mediators, and it is a key contributor to the nasal cycle. Persistent or exaggerated mucosal swelling occurs in allergic rhinitis, non-allergic rhinitis, and vasomotor disorders; over time, chronic inflammation may also induce remodeling of the bony framework [1,3,4].

Historically, surgical solutions to turbinate hypertrophy began with destructive approaches, including partial or total turbinectomy. While such procedures certainly enlarge the nasal cavity, they often lead to complications such as crusting, chronic nasal dryness, atrophic changes, and empty nose syndrome [1,5]. These experiences firmly established the importance of mucosal preservation and prompted the development of more refined, tissue-sparing techniques [1,6].

Today, multiple surgical options exist: submucosal resection, radiofrequency ablation, electrocautery, laser vaporization, microdebrider-assisted turbinoplasty, outfracture, and various forms of bony reduction [1,2,6,7,8,9]. Among structural techniques, endoscopic pyriform aperture turbinoplasty as described by Simmen and colleagues and mucosa-preserving powered inferior turbinoplasty as described by Wormald are particularly important, as they allow targeted correction of the skeletal component while preserving the mucosa [3,8,10,11]. At the same time, minimally invasive modalities such as radiofrequency and laser have become standard tools for treating mucosal hypertrophy [1,2,12].

Despite this evolution, there is still no universally adopted algorithm for choosing a specific technique or combination of techniques for an individual patient. The choice is often shaped by surgeon preference, institutional tradition, or equipment availability rather than a standardized, pathology-driven approach [1,2,5,6].

The purpose of the present work is to propose an evidence-informed decision-making framework for inferior turbinate surgery, explicitly linking the dominant anatomical and pathophysiological phenotype (cavernous/mucosal hypertrophy, bony hypertrophy, anterior nasal valve–turbinate conflict, or mixed hypertrophy) to a preferential surgical strategy. The framework is intended to support, not replace, comprehensive clinical assessment and shared decision-making, and requires prospective validation before it can be considered a formal clinical guideline. From a personalized medicine perspective, its central objective is to move beyond a “one-size-fits-all” approach to turbinate reduction and to tailor treatment according to the patient’s dominant structural phenotype, functional findings, comorbid inflammatory status, anatomical context, and individual preferences. Importantly, the proposed algorithm is not intended to assess the inferior turbinate in isolation. Inferior turbinate hypertrophy should always be interpreted within the broader anatomical context of nasal obstruction, including septal deviation, internal and external nasal valve morphology, lateral nasal wall stability, concha bullosa, sinonasal inflammatory disease, and rhinitis. Therefore, before any turbinate-directed procedure is selected, the algorithm requires a global endoscopic and functional assessment of the nasal airway. Turbinate surgery should be considered only when the inferior turbinate is judged to be a relevant contributor to obstruction, either alone or in combination with septal or valve abnormalities. In this sense, the inferior turbinate is treated not as an isolated anatomical structure, but as one component of an individualized nasal airway profile that should guide personalized surgical planning.

## 2. Materials and Methods

This study was designed as a structured narrative review aimed at developing a clinically applicable, phenotype-guided algorithm for the selection of inferior turbinate surgery. A narrative rather than systematic review design was chosen because of the marked heterogeneity of the available literature, including anatomical studies, histopathological investigations, imaging-based morphometric studies, surgical technique descriptions, randomized clinical trials, prospective and retrospective outcome studies, and expert-based surgical manuals. In addition, only a limited number of high-quality randomized controlled trials directly compare inferior turbinate techniques. Nevertheless, the review process was structured to improve transparency and reproducibility [1,2,5,6].

A literature search was performed in PubMed and Scopus from database inception to 1 February 2026. The search strategy combined terms related to the condition, anatomical substrate, diagnostic assessment, and surgical treatment. The following search terms were used alone and in combination: “inferior turbinate hypertrophy”, “inferior turbinate surgery”, “turbinoplasty”, “submucosal turbinoplasty”, “radiofrequency ablation”, “laser turbinoplasty”, “microdebrider-assisted turbinoplasty”, “powered inferior turbinoplasty”, “bony turbinate hypertrophy”, “inferior turbinate bone”, “pyriform aperture”, “nasal valve”, “nasal obstruction”, “rhinomanometry”, “acoustic rhinometry”, “computed tomography”, and “cone beam computed tomography”. Reference lists of relevant articles, standard rhinology textbooks, and surgical manuals were also screened manually to identify additional sources, including randomized and prospective clinical trials, histopathological studies, anatomical or biomechanical investigations, imaging studies, and validated classification systems [3,4,5,6,8,10,11,13].

Studies were considered eligible if they met at least one of the following criteria: (i) described the anatomy, histology, or radiological morphology of the inferior turbinate; (ii) evaluated clinical or functional outcomes after inferior turbinate surgery; (iii) compared mucosa-sparing and non-mucosa-sparing techniques; (iv) described surgical approaches targeting the mucosal or osseous component of the inferior turbinate; (v) addressed nasal valve or septal anatomy in relation to nasal obstruction; or (vi) proposed or validated an endoscopic, radiological, or clinical grading system relevant to inferior turbinate hypertrophy. Exclusion criteria were: studies not focused on inferior turbinate surgery or nasal obstruction, articles without accessible abstracts or sufficient methodological detail, non-English articles when no reliable translation was available, and purely technical reports without relevance to phenotype-based decision-making.

Data were extracted narratively and organized into predefined domains: anatomical target of treatment, diagnostic criteria, role of decongestion testing, role of endoscopy, role of CT/CBCT imaging, type of anesthesia, postoperative pain and morbidity, recovery profile, patient-reported outcomes, objective airflow outcomes, including rhinomanometry, acoustic rhinometry and peak nasal inspiratory flow (PNIF), complications, and durability of results. Particular attention was given to variables relevant to personalized treatment selection, including the dominant anatomical substrate, topographic distribution of hypertrophy, coexistence of septal or nasal valve abnormalities, patient-reported symptom burden, perioperative invasiveness, and feasibility of shared decision-making. Because the aim was to generate a practical decision-making framework rather than to perform a quantitative meta-analysis, no pooled effect estimates were calculated. Evidence was then synthesized to define clinically relevant phenotypes and to link each phenotype to a preferential treatment strategy. Four principal phenotypes of inferior turbinate hypertrophy were defined:

1. Predominantly cavernous/mucosal hypertrophy [1,2,14];
2. Predominantly bony hypertrophy [3,4,15];
3. Anterior nasal valve-turbinate conflict [10,16];
4. Mixed hypertrophy, where both mucosal and bony components coexist [1,4].

**Figure 1:**
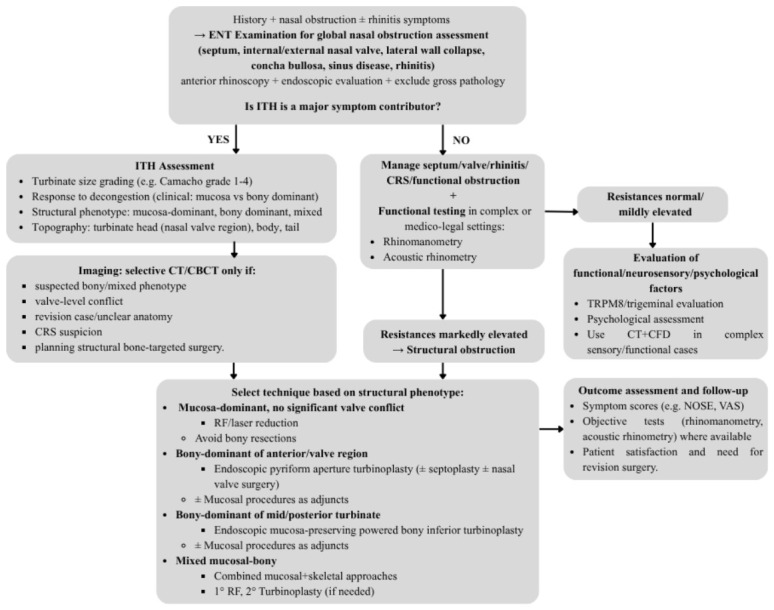
Phenotype-based algorithm for the management of inferior turbinate hypertrophy within the broader work-up of nasal obstruction. The algorithm integrates endoscopic assessment, functional testing and structural phenotyping (mucosal, bony or mixed hypertrophy; anterior nasal valve involvement; turbinate head/body/tail), and links each structural phenotype to a preferential surgical strategy (mucosa-preserving techniques, pyriform aperture turbinoplasty, endoscopic mucosa-preserving powered bony inferior turbinoplasty or combined approaches). Functional testing (rhinomanometry, acoustic rhinometry and, when available, PNIF) and, in selected cases, CT/CBCT with or without CFD modelling, as well as trigeminal/TRPM8 and psychological evaluation, are used to differentiate true structural obstruction from predominantly functional, neurosensory or psychogenic nasal obstruction and to avoid mismatched treatments. ITH: inferior turbinate hypertrophy; RF: radiofrequency; CT: computed tomography; CFD: computational fluid dynamics; NOSE: Nasal Obstruction Symptom Evaluation; VAS: visual analogue scale; TRPM8: transient receptor potential melastatin 8.

These categories are conceptually distinct from size-based grading systems developed for endoscopic assessment of inferior turbinate bulk, such as the validated four-grade, 25% increment classification proposed by Camacho et al. [13]. For each phenotype, the main contemporary techniques (pyriform aperture turbinoplasty, mucosa-preserving powered inferior turbinoplasty, radiofrequency ablation, laser turbinoplasty, bipolar electrocautery) were evaluated in terms of their anatomical target, expected benefits and limitations [1,2,3,5,6,8,10,11,12]. This evaluation informed the development of the practical decision algorithm summarized in Figure 1. In parallel, the available “building blocks” of turbinate classification (endoscopic volumetric grading, CT-based descriptions of bony morphology, and the conceptual mucosal/bony/mixed distinction) were critically appraised and recombined into a clinically oriented structural framework intended to guide, rather than simply describe, technique selection.

To improve transparency regarding evidence selection, the main sources informing the proposed framework are summarized in Table 1. The table is intended as an evidence map rather than a formal risk-of-bias assessment, because the review includes heterogeneous sources such as randomized trials, systematic reviews, histopathological studies, imaging studies, and surgical technique descriptions.

## 3. Results

The proposed algorithm is shown in Figure 1. The first step of the algorithm is a global nasal airway assessment rather than an isolated turbinate evaluation. This includes anterior rhinoscopy and nasal endoscopy to assess septal deviation, internal and external nasal valve narrowing or collapse, lateral wall stability, concha bullosa, inflammatory mucosal disease, and inferior turbinate size, consistency, topography, and response to decongestion. When septal deviation or nasal valve dysfunction is the dominant cause of obstruction, turbinate surgery alone is not recommended; instead, turbinate treatment should be integrated into a broader functional surgical plan, such as septoplasty, nasal valve repair, or combined septoturbinoplasty when appropriate. Below, we describe the four principal phenotypes of turbinate hypertrophy and illustrate how each phenotype can guide individualized selection of the most appropriate surgical strategy.

### 3.1. Cavernous/Mucosal Hypertrophy

Cavernous or mucosal hypertrophy is by far the most frequent pattern in clinical practice [1,2]. It is typically associated with rhinitis, either allergic, non-allergic, or mixed, and is characterized by a soft, readily compressible turbinate with pronounced response to topical vasoconstrictors. Endoscopy often shows boggy, edematous mucosa, occasionally with polypoid changes. The underlying bone is usually normal in size [1]. Radiofrequency ablation (RF) and laser turbinoplasty have become the mainstay of surgical treatment for this phenotype.

Radiofrequency ablation produces controlled submucosal thermal injury, leading to gradual fibrosis and contraction of the venous sinusoids. The mucosal surface is largely preserved, and the procedure can usually be performed under local anesthesia in an outpatient setting. Prospective series have demonstrated significant improvement in nasal obstruction scores and acoustic rhinometry measures, with minimal discomfort and no relevant alteration of ciliary function or mucosal architecture [1,2,12]. Long-term follow-up suggests that results remain acceptable for many patients, although some degree of recurrence is not uncommon, particularly in those with ongoing allergic inflammation [1,2].

Laser turbinoplasty (using diode, CO_2_, KTP, or Nd:YAG lasers) offers precise, superficial ablation with excellent hemostasis. Laser energy can be applied in a linear or spot pattern, reducing the thickness of the mucosa and submucosa while preserving deeper structures. Comparative data, including randomized allocation to laser versus other techniques, demonstrate significant improvements in nasal patency and nasal respiratory function, even though submucosal resection with lateral displacement may provide greater long-term airflow gains and better restoration of mucociliary clearance and secretory IgA production [1,2,5,6].

Bipolar electrocautery can also be used to treat mucosal hypertrophy by causing thermal coagulation of tissue. However, compared to RF and laser, bipolar cautery and related electrocautery techniques tend to generate more collateral thermal damage, with higher rates of crusting and less favorable long-term functional outcomes in comparative series [1,2,5,6]. For this reason, in a modern algorithm it is generally considered a second-line option in this phenotype when RF or laser are not available.

Crucially, bony reduction techniques such as endoscopic mucosa-preserving powered bony inferior turbinoplasty or pyriform aperture turbinoplasty do not address the underlying pathophysiology in pure cavernous hypertrophy and expose the patient to unnecessary skeletal manipulation. In the algorithm, these techniques are therefore explicitly avoided in cases of purely mucosal disease.

### 3.2. Bony Hypertrophy

The osseous component of the turbinate can enlarge due to developmental changes or adaptive remodeling in response to chronic inflammatory stimuli or altered airflow patterns. Histomorphometric studies of compensatory hypertrophy in the setting of septal deviation have shown that turbinate bone can undergo a twofold increase in thickness, with comparatively minor changes in the mucosal layers, suggesting that bone expansion may be the dominant driver of turbinate bulk in at least some phenotypes [4]. On clinical examination, the turbinate remains firm and bulky even after decongestion, on endoscopy a prominent bony contour with relatively less mucosal swelling is visualized [1,4]. In this setting, CT or CBCT imaging can be helpful to delineate the osseous framework of the inferior turbinate, particularly when clinical examination and decongestion testing suggest a substantial bony component, when anterior valve-level conflict is suspected, in revision cases, or when concomitant sinonasal disease must be assessed. However, routine CT for all patients with inferior turbinate hypertrophy is not recommended. In straightforward mucosa-dominant hypertrophy with clear endoscopic findings and good response to decongestion, imaging is unlikely to change management and may add unnecessary cost and radiation exposure.

In this context, mucosal-only techniques are insufficient. While RF or laser may provide transient improvement by thinning the mucosal envelope, they cannot adequately restore airway space if the bony framework remains enlarged.

Mucosa-preserving powered bony inferior turbinoplasty is specifically designed for this situation. The procedure involves raising a mucosal flap, exposing the turbinate bone, and selectively resecting the osseous structure under endoscopic guidance. The mucosa is then redraped, maintaining a lining over the remaining turbinate segment and preserving physiological function while widening the nasal airway [3,11].

In the anterior part of the nasal cavity, bony hypertrophy may also manifest as an encroachment of the turbinate head on the pyriform aperture and nasal valve. Here, pyriform aperture turbinoplasty is particularly useful. By releasing and reshaping the turbinate attachment at the pyriform rim, this technique directly addresses anterior structural stenosis, often in conjunction with septal or nasal valve surgery [8,10].

In the algorithm, therefore, mucosa-preserving powered bony inferior turbinoplasty is recommended for posterior and mid-portion bony hypertrophy, and endoscopic pyriform aperture turbinoplasty for anterior bony conflict. RF and laser are considered inadequate as standalone procedures in this phenotype and should be reserved only as adjuncts when residual mucosal hyperplasia remains.

### 3.3. Anterior Nasal Valve-Turbinate Conflict

The nasal valve region is the narrowest portion of the nasal airway and exerts a major influence on total nasal resistance. Any encroachment of the inferior turbinate into this region can cause significant obstruction even when the rest of the nasal cavity is relatively open. This anterior valve-turbinate conflict may result from a large turbinate head, a narrow pyriform aperture, a deviated septum, or a combination of these factors [1,10,16]. Clinically, patients often report obstruction that is worse during inspiration, sometimes with external nasal sidewall collapse. On endoscopy, the turbinate head appears to contact or closely approach the side of the nasal valve, leaving little or no free space.

In such cases, pyriform aperture turbinoplasty is the technique of choice. Its key features include precise dissection and reduction in the turbinate head at the pyriform aperture, widening of the anterior nasal cavity, preservation of mucosal coverage and potential synergy with concomitant septoplasty and functional nasal valve procedures [8,10].

Importantly, mucosa-focused techniques (RF, laser, bipolar) that do not modify the skeletal insertion of the turbinate head will generally fail to resolve the valve-level conflict. In the algorithm, these techniques are thus considered inappropriate as standalone treatments in this phenotype.

### 3.4. Mixed Hypertrophy

In clinical practice, many patients present with a combination of mucosal and bony changes. Mixed hypertrophy is particularly common in individuals with long-standing rhinitis and structural deviation who have developed both cavernous hyperplasia and osseous remodeling [1,4]. The challenge in mixed hypertrophy is to correct both components without causing excessive trauma. The algorithm therefore recommends hybrid procedures, such as powered bony inferior turbinoplasty in addition to RF ablation, or pyriform aperture turbinoplasty coupled with laser turbinoplasty. In these combinations, the bony procedure restores skeletal dimensions, while RF or laser refines the mucosal envelope and addresses residual hypertrophy of the cavernous tissue. By sequencing the interventions appropriately and preserving as much healthy mucosa as possible, the surgeon can optimize airflow while minimizing long-term complications.

To support shared decision-making, the main techniques included in the algorithm were also compared in terms of practical perioperative variables, expected benefits, and limitations (Table 2). These factors are clinically relevant because the optimal procedure is not determined solely by anatomy, but also by patient preferences, tolerance for postoperative morbidity, need for rapid recovery, comorbidities, and availability of local or general anesthesia. This comparison is intended to facilitate personalized counselling by matching the expected invasiveness, recovery profile, and durability of each procedure with the patient’s anatomical phenotype and treatment priorities.

Because several studies directly compared different inferior turbinate procedures, the main comparative studies are summarized separately in Table 3. Overall, these studies show that most contemporary mucosa-preserving techniques improve nasal obstruction, but differ in invasiveness, perioperative morbidity, durability, and ability to address bony versus mucosal components.

## 4. Discussion

The proposed algorithm presented in this article aims to translate anatomical and physiological insights into a simple, structured clinical tool. Three principles emerge. The proposed framework is intrinsically aligned with the principles of personalized medicine. Rather than selecting a turbinate procedure solely on the basis of surgeon preference, equipment availability, or global turbinate size, it encourages individualized treatment selection based on the dominant pathological substrate, the topographic site of obstruction, the contribution of adjacent structures, objective and subjective functional findings, and patient priorities. In this model, the same symptom—nasal obstruction—may lead to different interventions depending on whether the main driver is reversible mucosal congestion, osseous enlargement, anterior valve-level conflict, or a mixed phenotype.

First, mucosal preservation is fundamental. The historical lessons of aggressive turbinectomy have clearly demonstrated that excessive tissue removal carries serious long-term consequences, including atrophic changes and empty nose syndrome [1,2,5,17]. Comparative clinical trials and prospective series show that mucosa-sparing approaches such as submucosal resection with lateral displacement, microdebrider-assisted turbinoplasty, and radiofrequency ablation can achieve substantial and durable improvements in nasal airflow with lower complication rates, less crusting, and minimal discomfort [1,2,5,6,7,12]. These observations are consistent with contemporary proposals that the ideal inferior turbinate operation should be as minimally invasive as possible, emphasize submucosal remodeling rather than extensive resection, be individually selected for each patient, and aim for long-term preservation of nasal physiology rather than short-lived symptomatic relief [17]. At the same time, caution is warranted against overly absolute statements: the well-known “turbinate paradox” reminds us that a considerable number of patients have undergone partial or even extensive turbinectomy without developing clinically relevant symptoms. This suggests that over-resection, although clearly a risk factor, is probably not the sole determinant of postoperative dysfunction and that neurosensory, airflow-related, and patient-specific factors also likely contribute.

Second, anatomical specificity is paramount. The inferior turbinate is not a homogeneous structure; it encompasses anterior, middle, and posterior segments, each with distinct relationships to the septum, nasal valve, and lateral wall [3,10]. Histopathological data indicate that in some contexts, particularly compensatory hypertrophy contralateral to septal deviation, turbinate bone is the principal contributor to volume increase, with relatively minor mucosal changes, supporting bone-targeted interventions when skeletal expansion predominates [4]. Complementary CT morphometric data from asymptomatic adults show that, in the normal turbinate, the medial mucosal layer is the dominant contributor to bulk at both the anterior (valve) and posterior (choanal) ends. These normative findings reinforce the need to distinguish physiological mucosal predominance from pathological bony expansion when planning surgery [14]. Additional histopathologic evidence from chronic rhinosinusitis cohorts further supports a differential emphasis on mucosa versus bone: in patients with CRSwNP and CRSsNP, Patil et al. found that inflammatory changes and eosinophilic or lymphocytic infiltrates predominantly involve the mucosa and submucosa. These findings suggest that in polypoid disease mucosal resection with bone preservation is usually sufficient. In keeping with this mucosa-centered pathophysiology, a large Cone Beam Computed Tomography (CBCT) study by Razavi et al. demonstrated moderate correlations between inferior turbinate volume and maxillary sinus mucosal thickness on both the ipsilateral and contralateral sides, further supporting a shared inflammatory drive to mucosal hypertrophy across the sinonasal compartment [18]. At the same time, size-based endoscopic grading systems, such as the validated four-grade classification of Camacho et al., offer reproducible documentation of turbinate bulk that can complement phenotype-based decision-making [13]. Moreover, a systematic review of high-level evidence has shown that the correlation between subjective nasal patency and objective parameters derived from rhinomanometry and acoustic rhinometry is inconsistent [19]. Together, these data underline the need to interpret objective tests in conjunction with careful endoscopic assessment and patient-reported outcomes. For this reason, future studies and routine outcome reporting should combine patient-reported outcome measures, such as NOSE and VAS, with objective measures of nasal patency. Rhinomanometry and acoustic rhinometry provide useful functional and geometric information, whereas PNIF represents a simple, inexpensive, and repeatable office-based measure of global nasal airflow. PNIF has also been proposed as a useful tool for assessing outcomes after inferior turbinate radiofrequency/coblation procedures, particularly when combined with pre- and post-decongestion measurements. However, subjective and objective measures are not interchangeable: symptom perception is influenced by mucosal status, nasal cycle, trigeminal airflow perception, expectations, and psychological factors. Therefore, discordance between NOSE/VAS and rhinomanometry, acoustic rhinometry, or PNIF should not be interpreted as failure of either assessment method, but as evidence that multidimensional evaluation is required.

Third, the algorithm helps avoid mismatched treatments. Applying RF to a rigid, bony hypertrophy or performing a mucosal-only reduction where the anterior valve is structurally narrowed will predictably yield suboptimal results. Conversely, performing extensive skeletal reductions in purely mucosal disease is unnecessarily invasive. By clearly mapping each phenotype to a recommended and non-recommended set of techniques, the algorithm discourages such mismatches (Figure 1).

A further implication of phenotype-guided selection is that surgical choice should be shared with the patient. Techniques differ not only in anatomical target, but also in anesthesia requirements, postoperative discomfort, crusting, recovery time, durability, and likelihood of revision. For example, office-based radiofrequency may be preferable in patients with mucosa-dominant hypertrophy who prioritize minimal invasiveness and rapid recovery, whereas powered submucosal or bony turbinoplasty may be more appropriate when durable correction of a structural component is required. Presenting these trade-offs explicitly can improve patient counselling and align the surgical plan with both anatomical findings and patient preferences. This patient-centered approach is particularly relevant in personalized medicine, where the technically “best” procedure is not necessarily the most appropriate for every patient unless it is aligned with symptom severity, risk tolerance, recovery expectations, comorbidities, and long-term goals.

Fourth, turbinate surgery must be understood within the broader context of nasal function and adjacent anatomical structures. Septal deviation, concha bullosa, internal and external nasal valve narrowing, lateral wall collapse, and sinonasal inflammatory disease may each independently contribute to nasal obstruction and may also modify the apparent role of inferior turbinate hypertrophy. For this reason, the algorithm should not be interpreted as a stand-alone turbinate pathway, but as a turbinate-specific module embedded within a comprehensive nasal obstruction assessment. In many patients, optimal results are achieved only when turbinate surgery is combined with septoplasty, nasal valve repair, or treatment of inflammatory sinonasal disease [16]. Conversely, when septal deviation or valve collapse is the dominant mechanism of obstruction, isolated turbinate reduction may be insufficient and potentially misleading. Although a detailed discussion of these combined procedures is beyond the scope of this article, the algorithm is designed to be easily integrated into more global surgical planning. In patients with sleep-disordered breathing, such integrated management can have implications beyond nasal airflow alone, as several studies suggest that appropriately selected nasal and turbinate surgery may alleviate snoring, improve tolerance of positive airway pressure therapy, and reduce required pressure levels [17].

**Figure 2:**
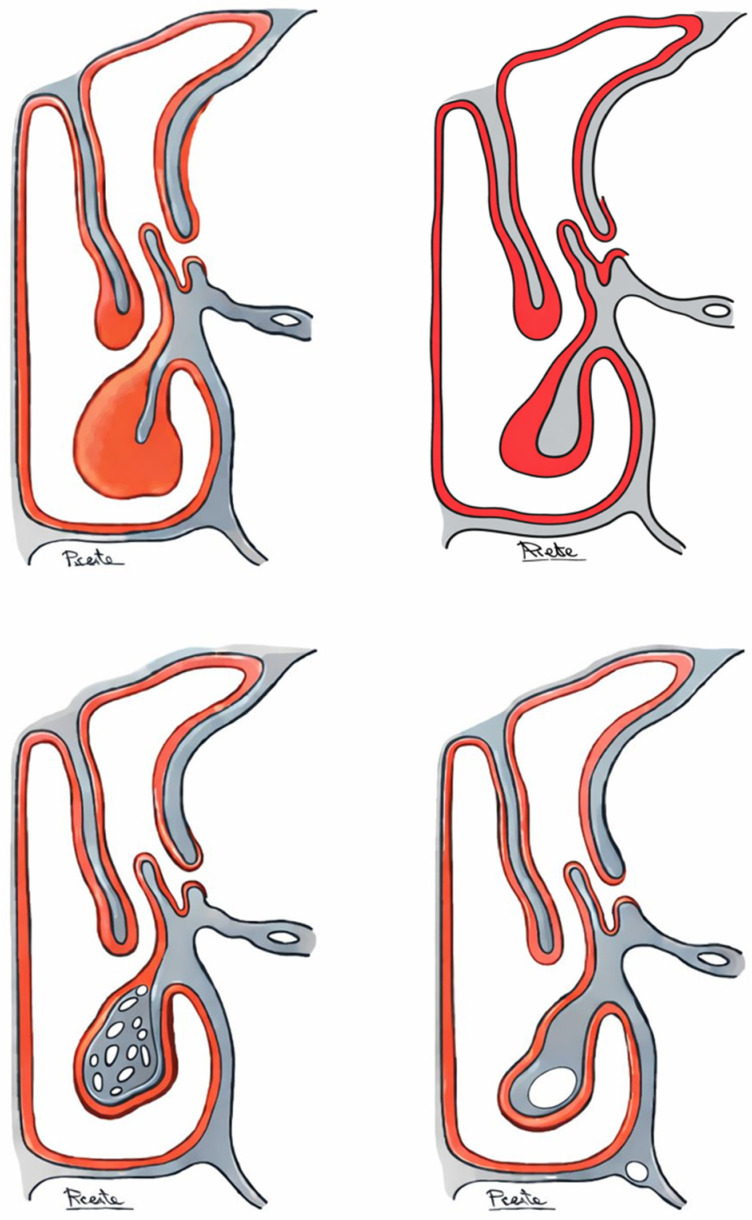
Schematic representation of the CT-based morphological classification of the inferior turbinate bone. Coronal views depict the four principal types described by Uzun et al. [15]: lamellar type (slender, plate-like bone), compact type (globular, thickened bone), combined type (showing both lamellar and compact components), and bullous type (pneumatized bone). The illustration was created by the graphic designer Andrea Presta and is based on the original radiologic classification; it is included here to facilitate phenotype-based selection of inferior turbinate surgery.

From the practical and classification standpoint, the current landscape remains fragmented. Endoscopic volumetric grading systems, such as the four-grade scale proposed by Camacho et al. [13], reliably quantify overall turbinate bulk but are essentially blind to the relative contribution of bone and mucosa and to fine topographic anatomy of the turbinate head, body, and tail. CT-based descriptions of bony morphology, including the lamellar, compact, combined and bullous types described by Uzun et al. [15], offer an elegant morphological taxonomy and a more detailed view of the osseous framework (Figure 2). In parallel, the conceptual tripartition into mucosal, bony, and mixed hypertrophy is clinically intuitive and supported by histological and imaging data, even if it lacks shared quantitative criteria (for example, percentage of bony versus mucosal volume), a uniform terminology, and explicit integration of the nasal valve segment, particularly the turbinate head region. Notably, this threefold subdivision has also been explicitly adopted in sleep-disordered breathing cohorts, underscoring its pragmatic value [17]. Quantitative CT measurements and three-dimensional reconstructions are promising research tools.

Emerging technologies should also be acknowledged. Piezo-assisted turbinoplasty has been proposed as a way to perform controlled bony weakening or outfracture while minimizing uncontrolled fracture lines and bleeding. In a prospective randomized study performed during septorhinoplasty, piezo-assisted turbinoplasty combined with bipolar coagulation achieved improvements in NOSE, rhinomanometry, and acoustic rhinometry comparable to partial turbinectomy, with minimal bleeding and shorter operative time [20]. Blue laser technology has also recently been introduced as an office-based treatment for inferior turbinate hypertrophy. Early pilot data show significant improvements in NOSE and VAS scores and good procedural tolerance, but the available evidence remains preliminary and objective outcome data are still limited [21]. These techniques may eventually fit within the proposed phenotype-based framework: piezo-assisted approaches may be most relevant for selected bony or mixed phenotypes requiring controlled lateralization, whereas blue laser may represent another minimally invasive option for mucosa-dominant hypertrophy. However, at present they should be considered emerging modalities pending larger comparative studies with standardized subjective and objective outcomes.

Imaging should be used selectively and proportionately. Although CT and CBCT can provide valuable information on turbinate bone morphology, septal anatomy, sinonasal disease, and anterior nasal valve relationships, the routine use of imaging in all patients with inferior turbinate hypertrophy is not justified. From a cost-effectiveness and resource-utilization perspective, imaging should be reserved for cases in which it is expected to modify management: suspected bony or mixed hypertrophy, anterior valve–turbinate conflict, revision surgery, unclear endoscopic findings, suspected chronic rhinosinusitis, or planning of structural bone-targeted procedures. Conversely, in uncomplicated mucosa-dominant hypertrophy with a clear response to decongestion, treatment can usually be planned on the basis of history, endoscopy, and functional assessment without mandatory CT.

Future research should focus on prospective validation of phenotype-guided decision-making. Ideally, multicenter studies should compare algorithm-guided treatment selection with usual surgeon-directed decision-making and should stratify patients according to mucosal, bony, anterior valve-related, and mixed phenotypes. Outcomes should be collected using standardized patient-reported measures, such as NOSE and VAS, together with objective measures including rhinomanometry, acoustic rhinometry, PNIF, and selective imaging-based morphometry. Follow-up should be sufficiently long to evaluate durability, recurrence, crusting, dryness, revision surgery, and patient satisfaction. Future trials should also report perioperative variables relevant to shared decision-making, including anesthesia type, postoperative pain, time to return to normal activity, need for packing, complications, and cost. Finally, emerging technologies such as piezo-assisted turbinoplasty, blue laser, refined CT/CBCT morphometry, and CFD-based surgical planning should be evaluated within the same phenotype-based framework to determine whether they provide measurable advantages over established techniques. Ultimately, such studies could support the development of validated personalized treatment pathways in rhinology, in which diagnostic phenotype, patient-reported burden, objective airflow impairment, and patient preference are integrated into a reproducible surgical decision model.

This study has several limitations. First, although the review process was structured, this was not a systematic review or meta-analysis, and therefore selection bias cannot be fully excluded. Second, the proposed phenotypes are derived from anatomical, histopathological, imaging, and surgical literature, but they have not yet been prospectively validated as a classification system. Third, the algorithm does not provide absolute indications; rather, it should be applied within a comprehensive nasal obstruction assessment that includes septal deviation, nasal valve anatomy, inflammatory disease, patient symptoms, and patient preferences. Fourth, the available literature does not consistently report comparable perioperative outcomes such as anesthesia type, postoperative pain, crusting, recovery time, or patient-reported outcome measures, limiting direct comparison between techniques. The cost-effectiveness of phenotype-guided imaging and surgical selection remains to be formally evaluated. In addition, although the framework is designed to support personalized treatment selection, the relative weight of each personalization variable, such as phenotype, objective airflow impairment, symptom burden, comorbid rhinitis, recovery expectations, and patient preference, has not yet been formally quantified. Finally, because several emerging techniques such as piezo-assisted turbinoplasty and blue laser treatment have only recently been reported, their role in the proposed algorithm remains provisional and should be reassessed as comparative and long-term outcome data become available.

## 5. Conclusions

Inferior turbinate hypertrophy is a heterogeneous condition arising from variable involvement of cavernous mucosa, submucosa, bony framework, and nasal valve relationships. Proper management requires more than a generic decision to reduce the turbinates: it demands identification of the dominant pathological substrate and matching that substrate to an appropriate, tissue-preserving surgical technique. The algorithm described in this article provides a rational, evidence-informed, personalized, and phenotypically grounded approach to technique selection. It promotes the use of radiofrequency or laser in mucosal-dominant disease, structural techniques such as mucosa-preserving powered bony inferior turbinoplasty and pyriform aperture turbinoplasty in bony or valve-level obstruction, and combined interventions in mixed hypertrophy, building on the concepts, histopathological insights, and outcome data summarized in recent reviews and clinical studies [1,2,4,5,6,7,10,12,13,19]. By adhering to the principles of mucosal preservation, anatomical specificity, shared decision-making, and avoidance of mismatched procedures, the algorithm offers a practical path toward more consistent, patient-centered, and personalized turbinate surgery.

## Figures and Tables

**Table 1:** Evidence map of the main sources informing the proposed phenotype-guided framework.

Reference	Study Type/Source	Main Focus	Key Contribution to the Framework
Abdullah and Singh [1]	Comprehensive review	Techniques for inferior turbinate hypertrophy	Overview of contemporary surgical options, complications, and mucosal preservation
Zhang et al. [2]	Systematic review	Surgical interventions for inferior turbinate hypertrophy	Summary of comparative outcomes and heterogeneity of available evidence
Wormald [3]	Surgical manual	Endoscopic powered inferior turbinoplasty	Technical basis for mucosa-preserving bony reduction
Berger et al. [4]	Histopathological study	Compensatory hypertrophy in septal deviation	Supports the concept of bony-dominant hypertrophy
Passàli et al. [5,6]	Randomized/long-term clinical trials	Comparative turbinate techniques	Outcome differences among mucosal and more destructive techniques
Zhang et al. [2]	Review	Graduated surgical management	Supports stepwise technique selection and mucosal preservation
Kanesan et al. [7]	Metanalysis	Microdebrider-assisted inferior turbinoplasty	Supports mucosa-preserving reduction approaches
Simmen et al. [8,10]	CFD/surgical technique description	Pyriform aperture turbinoplasty	Supports anterior valve–turbinate conflict as a distinct surgical target
Min et al. [9]	CT outcome study	Outfracture outcomes	Supports imaging-based evaluation of structural change
Joniau et al. [11]	Comparative long-term study	Submucosal cautery vs powered reduction	Supports powered reduction for durable turbinate volume control
Harju et al. [12]	Comparative long-term study	Comparative turbinate techniques	Supports RF as a safe mucosa-preserving approach
Camacho et al. [13]	Validation study	Endoscopic turbinate grading	Provides reproducible volumetric grading, but not structural phenotyping
El-Anwar et al. [14]	CT morphometric study	Normal inferior turbinate measurements	Provides normative CT data on mucosa, bone, and airway width
Uzun et al. [15]	CT classification study	Inferior turbinate bone morphology	Provides radiological bony categories
Hwang et al. [16]	Clinical surgical study	Endoscopic septoplasty	Supports integration of septal pathology in global nasal obstruction planning
Choi et al. [17]	Review	Turbinate surgery in sleep-disordered breathing	Supports individualized, minimally invasive, physiology-preserving surgery
Razavi et al. [18]	CBCT morphometric study	Turbinate volume and sinus mucosal thickness	Supports the role of imaging-based volumetric analysis
André et al. [19]	Systematic review	Subjective–objective correlation	Supports combined subjective and objective outcome assessment

**Table 2:** Comparison of the main techniques for ITH treatment.

Technique	Main Anatomical Target	Preferred Phenotype	Typical Anesthesia	Postoperative Pain/Recovery	Main Advantages	Main Limitations/Risks
**Radiofrequency ablation**	Submucosal cavernous tissue	Mucosa-dominant hypertrophy	Usually local anesthesia; outpatient feasible	Mild pain; rapid recovery; limited crusting	Minimally invasive; mucosa-preserving; low morbidity; repeatable	Less effective for bony hypertrophy; recurrence possible in uncontrolled rhinitis
**Laser turbinoplasty**	Mucosa/submucosa	Mucosa-dominant hypertrophy, selected bleeding-risk patients	Local or general anesthesia depending on setting	Mild–moderate discomfort; crusting depends on energy settings	Precise ablation; good hemostasis	Risk of crusting or thermal injury if excessive; limited effect on bone
**Bipolar/electrocautery**	Mucosa/submucosa by thermal coagulation	Second-line mucosal hypertrophy	Local or general anesthesia	More crusting and discomfort than RF in many series	Simple; widely available; inexpensive	Greater collateral thermal injury; less favorable mucosal recovery
**Microdebrider-assisted/submucosal turbinoplasty**	Submucosal soft tissue ± limited bone	Mucosal or mixed hypertrophy	Usually general anesthesia, sometimes local with sedation	Moderate early discomfort; recovery generally longer than RF	Effective volume reduction; mucosal preservation	Bleeding risk; packing may be needed; requires equipment and surgical setting
**Mucosa-preserving powered bony inferior turbinoplasty**	Inferior turbinate bone with mucosal flap preservation	Bony mid/posterior hypertrophy	Usually general anesthesia	Moderate recovery; depends on extent of bone work	Directly addresses osseous hypertrophy; preserves mucosa	More invasive than RF/laser; bleeding/crusting risk; not indicated for pure mucosal disease
**Endoscopic pyriform aperture turbinoplasty**	Anterior turbinate head/pyriform aperture	Anterior valve–turbinate conflict	Usually general anesthesia	Moderate recovery; often combined with septal/valve surgery	Targets valve-level obstruction; useful for turbinate head conflict	Not indicated for isolated posterior or purely mucosal hypertrophy; requires careful patient selection
**Combined procedures**	Bone + mucosal envelope	Mixed hypertrophy	Usually general anesthesia	Recovery depends on extent of combined surgery	Addresses both components; tailored treatment	Risk of overtreatment if phenotype is not correctly defined

**Table 3:** Comparative studies of inferior turbinate surgical techniques.

Study	Compared Techniques	Outcome Measures	Main Findings
Passàli et al. [5]	Multiple techniques including submucosal resection, laser, electrocautery	Nasal respiratory function, mucociliary clearance, secretory IgA	Submucosal resection with lateral displacement showed favorable long-term functional results compared with more destructive approaches
Passàli et al. [6]	Randomized allocation to different turbinate treatments	Long-term nasal function and complications	More conservative/mucosa-preserving techniques had better functional profiles and fewer mucosal side effects
Joniau et al. [11]	Submucosal cauterization vs powered inferior turbinate reduction	Long-term symptom and functional outcomes	Powered reduction showed durable benefit compared with submucosal cauterization

## Data Availability

No new data was created in this study. This article is based on previously published literature, and data sharing is not applicable to this article.

## Abbreviations

CBCT, cone beam computed tomography; CFD, computational fluid dynamics; CRSsNP, chronic rhinosinusitis without nasal polyps; CRSwNP, chronic rhinosinusitis with nasal polyps; CT, computed tomography; IgA, immunoglobulin A; ITH, inferior turbinate hypertrophy; KTP, potassium titanyl phosphate; Nd:YAG, neodymium-doped yttrium aluminum garnet; NOSE, Nasal Obstruction Symptom Evaluation; RF, radiofrequency; TRPM8, transient receptor potential melastatin 8; VAS, visual analogue scale; PNIF: peak nasal inspiratory flow.

## References

[1] Abdullah B., Singh S. (2021). Surgical Interventions for Inferior Turbinate Hypertrophy: A Comprehensive Review of Current Techniques and Technologies. Int. J. Environ. Res. Public Health.

[2] Zhang K., Pipaliya R.M., Miglani A., Nguyen S.A., Schlosser R.J. (2023). Systematic Review of Surgical Interventions for Inferior Turbinate Hypertrophy. Am. J. Rhinol. Allergy.

[3] Wormald P.J. (2018). Endoscopic Sinus Surgery: Anatomy, Three-Dimensional Reconstruction, and Surgical Technique.

[4] Berger G., Hammel I., Berger R., Avraham S., Ophir D. (2000). Histopathology of the inferior turbinate with compensatory hypertrophy in patients with deviated nasal septum. Laryngoscope.

[5] Passàli D., Passàli F.M., Damiani V., Passàli G.C., Bellussi L. (2003). Treatment of inferior turbinate hypertrophy: A randomized clinical trial. Ann. Otol. Rhinol. Laryngol..

[6] Passàli D., Lauriello M., Anselmi M., Bellussi L. (1999). Treatment of hypertrophy of the inferior turbinate: Long-term results in 382 patients randomly assigned to therapy. Ann. Otol. Rhinol. Laryngol..

[7] Kanesan N., Norhayati M.N., Hamid S.S.A., Abdullah B. (2022). Microdebrider-assisted inferior turbinoplasty versus other surgical techniques. Acta Otorhinolaryngol. Ital..

[8] Simmen D., Sommer F., Briner H.R., Jones N., Kroger R., Hoffmann T.K., Lindemann J. (2015). The effect of “Pyriform Turbinoplasty” on nasal airflow using a virtual model. Rhinology.

[9] Min J.Y., Dhong H.J., Cho H.J., Chung S.K., Kim H.Y. (2013). Evaluation of inferior turbinate outfracture outcomes using computed tomography. Rhinology.

[10] Simmen D., Schmid N., Simmen D., Jones N. (2014). Pyriform turbinoplasty as an adjunct to nasal valve surgery. Manual of Endoscopic Nasal Surgery.

[11] Joniau S., Wong I., Rajapaksa S., Carney S.A., Wormald P. (2006). Long-Term Comparison Between Submucosal Cauterization and Powered Reduction of the Inferior Turbinates. Laryngoscope.

[12] Harju T., Numminen J. (2022). The Long-term Effect of Inferior Turbinate Surgery Techniques on Nasal Obstruction and Quality of Life. Ann. Otol. Rhinol. Laryngol..

[13] Camacho M., Zaghi S., Certal V., Abdullatif J., Means C., Acevedo J., Liu S., Brietzke S.E., Kushida C.A., Capasso R. (2015). Inferior turbinate classification system, grades 1 to 4: Development and validation study. Laryngoscope.

[14] El-Anwar M., Hamed A., Abdulmonaem G., Elnashar I., Elfiki I. (2017). Computed Tomography Measurement of Inferior Turbinate in Asymptomatic Adult. Int. Arch. Otorhinolaryngol..

[15] Uzun L., Ugur M.B., Savranlar A., Mahmutyazicioglu K., Ozdemir H., Beder L.B. (2004). Classification of the inferior turbinate bones: A computed tomography study. Eur. J. Radiol..

[16] Hwang P.H., McLaughlin R.B., Lanza D.C., Kennedy D.W. (1999). Endoscopic septoplasty: Indications, technique, and results. Otolaryngol.—Head Neck Surg..

[17] Choi J.H., Lee J.K., Cho S.H. (2018). Inferior Turbinate Surgery in Sleep-Disordered Breathing Patients with Nasal Obstruction: Principles and Various Techniques. Sleep Med. Res..

[18] Razavi M., Sharifishoshtari S., Afshari F., Rakhshan V. (2024). Associations between the Volume of Bilateral Inferior Turbinates with Ipsilateral and Contralateral Sinus Mucosal Lining Thicknesses in Various Ages and Sexes: A CBCT Study of 302 Individuals. Maedica.

[19] André R.F., Vuyk H.D., Ahmed A., Graamans K., Nolst Trenité G.J. (2009). Correlation between subjective and objective evaluation of the nasal airway. A systematic review of the highest level of evidence. Clin. Otolaryngol..

[20] Verkest V., Pingnet L., Fransen E., Declau F. (2022). Piezo-assisted Turbinoplasty Versus Partial Turbinectomy in External Septorhinoplasty: A Prospective Comparative Study in 100 Patients. Aesthetic Plast. Surg..

[21] Hamdan A.L., Hosri J., Yammine Y., Nawfal N., Kasty M., Abou Raji Feghali P., Ghzayel L., Alam E. (2024). Office-based blue laser therapy for inferior turbinate hypertrophy: A pilot study. Eur. Arch. Oto-Rhino-Laryngol..

